# Phytomedicinal properties of *Hygrophila schulli* (Neeramulliya)

**DOI:** 10.22038/IJBMS.2023.67965.14877

**Published:** 2023

**Authors:** Malshani Chathuranika Nissanka, Manjula Manoji Weerasekera, Ayomi Dilhari, Ranga Dissanayaka, Sajeewa Rathnayake, Gayan Kanchana Wijesinghe

**Affiliations:** 1 Department of Microbiology, Faculty of Medical Sciences, University of Sri Jayewardenepura, Nugegoda, Sri Lanka; 2 Department of Basic Sciences, Faculty of Allied Health Sciences, University of Sri Jayewardenepura, Nugegoda, Sri Lanka; 3 Department of Pharmacy and Pharmaceutical Sciences, Faculty of Allied Health Sciences, University of Sri Jayewardenepura, Nugegoda, Sri Lanka; 4 The Centre for Infectious Diseases and Phytochemical Studies, Faculty of Integrated Life Sciences, Quest International University Perak, Perak, Malaysia; 5 Bristol Dental School, University of Bristol, United Kingdom; 6 Department of Medical Laboratory Sciences, Faculty of Allied Health Sciences, University of Sri Jayewardenepura, Nugegoda, Sri Lanka

**Keywords:** Alkaloids, Anti-bacterial agents, Anti-infective agents, Anti-inflammatory agents, Anti-oxidants, Biodiversity, Plant extracts, Stigmasterol

## Abstract

*Hygrophila schulli* which is known as “Neermulli’’ in the vernacular is an herbaceous plant native to Sri Lanka. Ancient medicinal literature suggests the use of *H. schulli* whole plant or its parts for the treatment of different communicable and non-communicable diseases including diabetes mellitus and tuberculosis. Active constituents and secondary metabolites including alkaloids, tannins, steroids, proteins, flavonoids, and glycosides are identified to possess antimicrobial, antitumor, antioxidant, hepatoprotective, anthelmintic, nephroprotective, antidiabetic, anticataract, anti-inflammatory, anti-nociceptive, hematopoietic, diuretic, antiurolithiatic, antipyretic, neuroprotection, and anti-endotoxin activities. In this review, we reviewed clinical studies, patents, and analytical studies from the earliest found examples from 1886 to the end of 2021. We critically analyzed and attempt to summarize the information based on bioactivities and chemical composition of *H. schulli* plant extracts which will be of future use for researchers in this field.

## Introduction

Natural medicines are considered the primary sources of medicinal agents over centuries in various cultures with social norms, ethical values, and traditional customs. Herbal medicine is the pioneering branch of natural medicine because over 90% of natural medicines are plant related. Therefore, the contribution of herbal medicine to the current pharmacopeia is significant. Approximately 80% of the global populace relies on traditional herbal medicine for primary health care (1) due to various reasons including its safety, long pharmacopeial history, availability and affordability, high efficacy, and low side effects. Herbal medicines are known to have a wide range of biological activities including anti-oxidant, anti-inflammatory, anticancer, and antifibrotic activities, etc., and are widely used as alternative or complementary medicines for the prevention and treatment of diverse disease conditions (2). 

Sri Lanka is considered a biodiversity hotspot with a high degree of endemism. Therefore, plants growing on this tiny island have unique features including morphological and biological features. The use of herbs as medicinal agents in Sri Lanka has been enriched by several medicinal systems including traditional folk medicine, Ayurveda, Unani, and Siddha. The use of herbal medicine in traditional and Ayurveda medicinal systems has over 3000 years of documented history.

*Hygrophila schulli* is one such plant, indigenous to Sri Lanka (3), which is commonly used in both traditional and ayurveda systems to treat many disease conditions. In Sri Lanka, it is known as “Neeramulliya” or “Niramulli”. 

In this review, the various scientifically proven photochemical properties of *H. schulli* are discussed to strengthen the use of this valuable plant as herbal medicine. The plant’s common habitat is moist or wet places, especially in Asia and African regions (4). 

Roots, leaves, flowers, seeds, and also the whole plant of *H. schulli* are used for treatment (5). The bioactivity and chemical analysis of various extracts of *H. schulli* was carried out by scientists, globally. Aqueous, alcoholic, or hydro-alcoholic extracts of *H. schulli *whole plant and various parts of the plant independently possess many important bioactivities, including anti-oxidant (6), anticancer (7), antibacterial (8), antifungal (9), hepatoprotective (10), anti-inflammatory (4), and anti-nociceptive (11) activities. 

In Ayurveda, *H. schulli *leaves and young stems are frequently used for the treatment of dysentery, edema, inflammation, cough, joint pains, bacterial infections, rheumatism, renal calculi, and other renal diseases, hepatic diseases, and microbial infections such as gonorrhea and urinary tract infections, etc. (12). It has been found that *H. schulli *mainly contains alkaloids, lupeol, stigmasterol, isoflavone, glycoside and uncharacterized bases, etc. (13). However, the chemical constituents of the plant extracts are varying depending on the geographic origin of the plant, the solvents to be used for extractions and conditions to be applied, etc. (5). Therefore, contradictory data have been reported regarding the phytochemical composition of the different extracts of *H. schulli*. This review analyses the available scientific evidence on different bioactivities of *H. schulli* extracts as an alternative therapeutic strategy for various lifestyle diseases that require lifelong pharmaceutical medication to raise the quality of individuals’ lives.

***Botanical Description of Hygrophila schulli*** ***species***

*H. schulli *belongs to Kingdom: Plantae; Phylum: Tracheophyta; Class: Magnoliopsida; Order: Scrophulariales; Family: *Acanthaceae*; Genus: *Hygrophila*; Species: *H. schulli *(5). *H. schulli* is widely distributed and used as a folk medicine in South Asia (i.e., Bangladesh, India, Nepal, Pakistan, and Sri Lanka), China, Myanmar, Malaysia, Burma, and Tropical Africa (14). According to the flora of *H. schulli*, it is recognized with a variety of synonyms such as *Asteracantha longifolia (L.) Nees, Bahel schulli Buch.- Ham, Barleria auriculata Schumach, Barleria longifolia L, Hygrophila longifolia (L.) Kurz, Hygrophila spinosa T.Anderson, *and* Hygrophila spinosa T. Anders *(15)*.* In Sri Lanka, *H. schulli* is found in the dry zone and ditches and marshy lands in the low country (3). 

**Figure 1:**
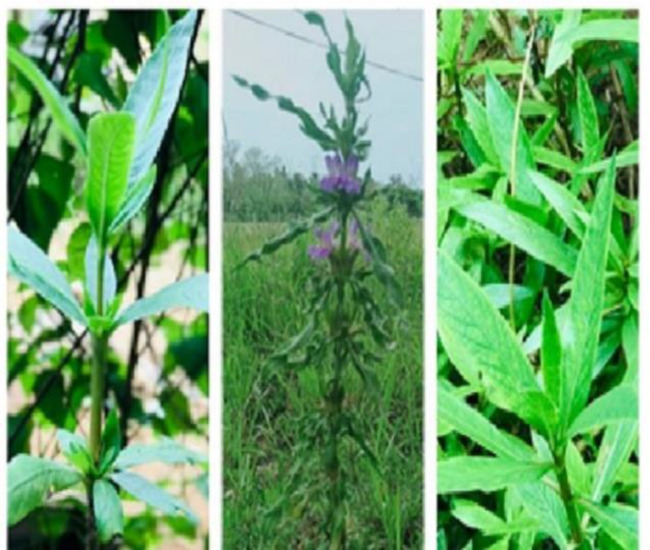
Entire plant of *Hygrophila schulli*

*H. schulli* plant is an annual, approximately 1.5 m in height spiny herb (12). In general, it contains eight leaves and six spines at each node. The leaves are sessile, whorled, and have undulating margins (5). Flowers arise from October to December and are 2-3 cm long, purple-blue with a 4-lobed calyx (11,14). There are about 4-8 orbicular seeds on the hard retinacula which are 7.5 mm in length and 0.3 mm across with a linear-oblong capsule (14). According to the International Union for Conservation of Nature  (IUCN) 2011, *H. schulli* species has been identified under the least concern group (16)(Figure 1).

***Bioactivities of Hygrophila schulli***

*Antimicrobial Activity of H. schulli*

Many studies have been conducted to determine the antibacterial and antifungal activities of *H. schulli* extracts. We have noted that the antimicrobial activity of a given extract depends on various factors such as extraction method, solvents used, parts of the plant used to extract phytochemicals, and especially, the mode of antimicrobial action and the effective concentrations depending on the constituents present in the extract and their abundance. 

The multidrug resistance (MDR) of pathogenic microbial species is becoming an emerging problem in the healthcare setting. The emergence of MDR is leading to the identification of novel therapeutic alternatives, also studies on their clinical applicability, toxicity, and mode of action are becoming a necessity when the health effects of MDR are concerned (17). In this case, plant derivatives act as a potential drug candidate with high potency, low toxicity, wide availability, and accessibility (18). Importantly, pathogens usually do not develop MDR against plant-based antimicrobials. Furthermore, many plant species are rich in secondary metabolites with antimicrobial activity. Thus, natural herbal products based on novel antimicrobial drug discovery are exhibiting great success (19, 20).

Table 1 summarizes the findings of some studies done to detect the antimicrobial activity of *H. schulli* plant extract. 

***Anti-biofilm Activity of H. schulli***

Even though many studies show the antimicrobial activity of *H. schulli *extracts, their anti-biofilm activity is not well studied yet. Biofilm is a complex arrangement of different microorganisms. Microbial biofilms are cellular aggregates covered with exopolymeric substances (EPS) attached to both biotic surfaces such as host cells and abiotic surfaces such as medical devices. Microbial biofilms are inherently resistant to routine antimicrobial agents compared to planktonic counterparts, due to their molecular contents such as eDNA and exoenzymes, reduced diffusion of antimicrobial agents through the biofilm matrix, persistent cellular content, and limited nutrients and oxygen (29). Global statistics reveal that the majority of chronic human infections are biofilm related. Therefore, it is very important to investigate effective antimicrobial agents with anti-biofilm activity. In this case, phytotherapeutic approaches become a potential candidate. Therefore, future studies were recommended to identify plant species with anti-biofilm effects. 

***Anti-oxidant and free radical scavenging activities of H. schulli***

Anti-oxidants are emerging as therapeutic and prophylactic agents which scavenge free radicals like Reactive Oxygen Species (ROS) and reduce the damage caused by them (30). Free radicals have the potential to destroy healthy cells of the body by damaging their structure and functions (5). Free radicals are accountable for causing a tremendous number of diseases and health conditions which include cardiovascular diseases, cancers, Alzheimer’s disease, neural disorders, mild cognitive impairments, alcohol-induced liver disease, Parkinson’s disease, ulcerative colitis, atherosclerosis, and aging (5, 31). Oxidative stress which is an imbalance between oxidants and anti-oxidants leads to many biochemical changes. Recently, interest in searching for naturally occurring anti-oxidants has increased considerably due to the adverse side effects of synthetic anti-oxidants, such as hepatotoxicity, carcinogenicity, and nephrotoxicity (32).

The medicinal plant, *H. schulli* is commonly prescribed by ayurvedic practitioners as an anti-oxidant and free radical scavenging agent. Many studies have been conducted to determine the anti-oxidant and free radical scavenging activity of different parts of *H. schulli*. Those studies and their findings were summarized in Table 2.

***Hepatoprotective activity of H. schulli***

The liver is a vital organ of the human body that plays a pivotal role in regulating various physiological activities in the body. It regulates numerous vital functions, such as metabolism, storage, and secretion. It has a huge capacity to detoxicate toxic substances such as caffeine and alcohol and synthesize useful substances such as blood clotting factors. Therefore, damage to the liver by hepatotoxic agents is of huge consequence (33). Still, there are no effective drugs in modern medicine, to regenerate hepatic cells and stimulate liver functions (34). In traditional and ayurvedic medicine, many medicinal plant preparations are recommended for the treatment of liver disorders and often offer significant relief (35). Scientific evidence reveals that the extractions of *H. schulli* achieve great success in the treatment of hepatic disorders (Table 2). 

***Anti-diabetic activity of H. schulli***

There has been an exponential increase in the occurrence of diabetes in modern society, due to the recent lifestyle deviations and shift towards excessive urbanization. Globally more than 420 million people are estimated to be suffering from diabetes mellitus. Indigenous plant-based treatments are greatly useful due to the high cost and possible toxicity associated with Western medicine. However, crude drugs derived from plants must also be subjected to extensive phytochemical analysis to ensure efficacy and safety and to develop sustainable, safe, and marketable therapeutic drugs (36). With the high occurrence of diabetics in society, scientists pay attention to finding novel ways to treat and control diabetes and associated complications, especially with the use of phytomedicinal components.

Due to extensive folk medicine systems like Ayurveda and Siddha, Sri Lanka is blessed with a wealth of ancestral knowledge about medicinal plants like *H. schulli*. Extracts of *H. schulli* hold greater promise for a country like Sri Lanka where there is an unprecedented rise in patients suffering from diabetes mellitus.

Table 2 summarizes the findings of *in vitro *and* in vivo* studies on the anti-diabetic activity of *H. schulli* plant extracts. 

***Anti-nociceptive and anti-inflammatory activities of H. schulli***

The management of pain and inflammation with routine opioid analgesics and non-steroidal anti-inflammatory drugs is recently encountering severe adverse effects like tolerance, dependence, and gastrointestinal problems (4, 37). Currently, traditional medicinal plants are widely used as alternative remedies for the treatment of pain and inflammation. *H. schulli* is one of the traditionally used medicinal plants for the treatment of pain and inflammatory conditions (11). Moreover, most of the studies show that the seed extract of *H. schulli* possessed significant anti-nociceptive and anti-inflammatory activities (Table 2). *H. schulli* leaves macerated in alcohol are used for the management of headaches while fresh leaves of *H. schulli* are used against inflammatory conditions in the skin (4). 

***Anti-cancer activity, anti-tumor activity, and cytotoxicity of H. schulli***

According to the global cancer statistics in 2020, it is estimated that there were 19.3 million new cancer cases and 10.0 million cancer deaths in 2020. Sung *et al.* in 2021 revealed female breast (11.7%), lung (11.4%), prostate (7.3%), non-melanoma of skin (6.2%), colon (6.0%), stomach (5.6%), liver (74.7%), rectum (3.8%), and cervix uteri (3.1%) as the commonest sites for the development of malignancies (52). 

Due to the adverse effects arising with the use of common anti-cancer drugs, traditional medicinal plants have been investigated as an alternative source of therapeutic agents which potentially had lesser adverse side effects. *H. schulli* is one such plant where a crude extract of the plant exhibited extensive anti-cancer and anti-tumor activities against different types of cancers and different experimental models. According to Uddin *et al*. in 2011, SK-BR-3, MCF7, HCT 116, SGC-7901, and Hs605T are some cell lines affected by the anticancer activity of *H. schulli* plant extract (1). 

The use of natural medicinal plants as therapeutic agents is becoming more popular in the modern world. Toxicological assessment of a natural product is an important aspect that should be concerned before the clinical application (19). Modern scientists conduct studies on the cytotoxic effects of *H. schulli *to ensure the safe use of this important phytochemical agent. 

Several toxicological studies have proved that at a dose of 2000 mg/kg body weight (BW), *H. schulli* plant extracts had not expressed any significant changes in the biochemistry parameters, weight of the internal organs, body weight, and food and water consumption of experimental animal models (53). Although *H. schulli* plant extracts were found to have no cytotoxic effect against normal cells, they produced selective cytotoxic activity towards tumor cells at a range of IC50 0.22–1.6 mg/ml (1, 13). Importantly, some controversial findings were reported by various scientists on the toxicological characteristics of *H. schulli* extracts. The extraction method, solvents used, geography and botanical properties of the plant, and environmental factors can influence such discrepancies.

Table 3 summarizes the findings of* in vitro *and* in vivo* studies on anti-cancer, anti-tumor activities, and cytotoxicity of *H. schulli* plant extracts.

***Chemical composition of Hygrophila schulli***

Phytochemicals obtained from medicinal plants play a significant role in medicinal science to cure or hinder critical health conditions. Researching phytochemical screening has created a new bridge between conventional and modern drug compounds (5). Phytochemical screening is usually carried out on the whole plant or sometimes on a particular part of the plant (stem, leaves, roots, seeds, etc.) to get the desired bioactive compounds (57). 

Phytochemically, the extracts of the whole plant generally contain phytosterols, carbohydrates, tannins, terpenoids, flavonoids, and sterols. Apigenin-7-glucoside and apigenin-7-O-glucuronide were isolated from the flowers (58). According to the results of the study conducted by Phalnikar *et al*, oil from the seeds of *H. schulli* consists of uronic, stearic, palmitic, oleic, and linoleic acids. Alkaloids, tannins, steroids, proteins, carbohydrates, flavonoids, fats, and oils were isolated from the roots (59). Moreover, the leaves of *H. schulli *show the presence of carbohydrates, alkaloids, steroids, proteins, flavonoids, glycosides, phenolic compounds, tannins, fats, and oils (58).

Phytochemical constituents and their relative abundance play a major role in the determination of the biological activity and efficacy of phytomedicinal preparations. The chemical composition of the extract depends on various facts such as extraction method, solvents used, and parts of the plant used to extract phytochemicals.

Ethnomedicinally it has been reported that the plant *H. schulli* is used for the treatment and prevention of various human disease conditions such as arthritis, allergies, anemia, cancers, hypertension, body pain, constipation, cough, renal dysfunction, diarrhea, dysentery, fistula, edema, gall stones, urinary calculi, kidney stone, inflammations, leprosy, jaundice, liver disorder, skin disease, leucorrhoea, rheumatism, tuberculosis, malaria, urogenital disorder, and venereal diseases.

The anticancer activity of *H. schulli* is due to the presence of phenols and flavonoids. Phenolic compounds reduce the formation of pre-cancerous cells. Flavonoids may protect DNA from oxidative damage, inhibit carcinogen activation, activate carcinogen detoxifying systems, and interfere with the development of malignant tumors (55, 60).

Ethanolic extract of *H. schulli* leaf demonstrated anti-inflammatory properties. This may be due to the hyaluronidase inhibition property of the extract. Flavonoids and tannins are some such hyaluronidase inhibitory compounds present in *H. schulli* extracts. Hyaluronidase is an enzyme that degrades β1, 4 glycosidic linkage of hyaluronic acid which leads to the activation of pro-inflammatory cytokines. Whenever there is inflammation, tissue permeability is enhanced by the hyaluronidase and spread the inflammatory responses around the affected organ. Furthermore, secondary metabolites such as stigmasterol, lupeol, and lup-20 (29)-ene-3β,23-diol also contribute to the anti-inflammatory activity of *H. schulli* leaf extract (4).

The availability of flavonoids, sterols, terpenoids, aliphatic esters, and botulin among the plant constituents contributes to the protective effect of *H. schulli* against hepatotoxins. The flavonoids are known antiperoxidants, anti-oxidants, and free radical scavengers leading to hepatoprotection (3).

Phytoconstituents such as tannins, flavonoids, alkaloids, phenolics, compounds, and saponins are responsible for the antibacterial effect. Irreversible complexes are formed by tannins with proline-rich proteins, resulting in the inhibition of cell wall synthesis. Lipophilic flavonoids exhibit antimicrobial activity by reacting with lipid components and disrupting microbial cell membranes.

Seeds of *H. schulli* are a potential source of anti-oxidants that prevent DNA damage. The presence of flavonoids and phenolic compounds like quercetin and tricin in *H. shulli* seeds prevents the production of ROS by complexing cations, indicating the protection of DNA (16).

**Table 1:** *In vitro* studies on anti microbial activity of *Hygrophila schulli* plant extracts

Property	Extraction		Tested organisms	Activities/ Remarks	Reference/s
	Part of the plant	Solvent(s)			
Antibacterial Activity	Leaves	Ethanol	*Escherichia coli, Klebsiella pneumoniae, Citrobacter divergens,* *Enterococcus faecalis, *and* Serratia marcescens*	Growth inhibited in *E. coli* and *K. pneumoniae.* No apparent inhibitory activity against *E. faecalis, C. divergens, *and* S. marcescens*	(21)
		Methanol	*Bacillus subtilis, Mycobacterium smegmatis, Pseudomonas aeruginosa, Salmonella gallinarum, *and* Staphylococcus aureus*	Growth inhibited in *Bacillus subtilis*,* M. smegmatis*, and *S. aureus.* No activity against *P. aeruginosa *and* S. Gallinarum*	(22)
			*Escherichia coli, Enterobacter aerogenes, Proteus vulgaris, Proteus mirabilis, Pseudomonas aeruginosa, Staphylococcus aureus, *and* Burkholderia pseudomallei*	Growth inhibited in *E. aerogenes, P. aerogenes, S. aureus, *and* B. pseudomallei* No activity against *E. coli, P. vulgaris, P. Mirabilis, *and* P. aeruginosa.*	(23)
		Chloroform, petroleum ether, alcohol and water	*Escherichia coli, Pseudomonas aeruginosa, Staphylococcus aureus, *and* Bacillus subtilis*	Chloroform and alcoholic, aqueous, and petroleum ether extracts exhibited significant-, moderate- and least-growth inhibitory activities, respectively.	(24)
		Water	*Bacillus cereus, Staphylococcus aureus, Streptococcus pneumonia, Escherichia coli, Pseudomonas aeruginosa, Klebsiella pneumonia, Salmonella typhi, Proteus vulgaris, *and* Shigella flexneri*	Growth inhibited at 200 µg/ml of the extract	(25)
	Stem	Ethanol	*Staphylococcus aureus, Escherichia coli, *and *Pseudomonas aeruginosa*	Exhibited significant growth inhibition against *S. aureus, *and less activity against *P. aeruginosa *and *E. coli.*	(26)
	Whole plant	Diethyl ether	*Staphylococcus epidermidis, Escherichia coli, Vibrio cholerae, Enterococcus fecalis, *and *Salmonella typhi*	Exhibited significant growth inhibition	(27)
		Ethanol	*Escherichia coli, Vibrio cholerae, Salmonella typhimirium, Bacillus cereus, Proteus mirabilis, Shigella dysenteriae, *and* Staphylococcus aureus*	Exhibited growth inhibition	(8)
		Water	*Escherichia coli*	Significant antibacterial activity against *E. coli* strains by steam extract. The MIC against both *E. coli* ATCC 25922 and the clinical isolates: 0.6 g/ml.	(28)
Antifungal	Leaves	Ethanol	*Candida albicans, Microsporum canis, *and* Trichophyton mentagrophytes*	Exhibited significant growth inhibition against *M. canis *and* T. mentagrophytes* Minimum activity against *C. Albicans*	(26)
			*Aspergillus niger, Aspergillus flavus, Aspergillus fumigatous, Rhododendron inducum, *and* Fusarium *spp.	Exhibited significant growth inhibition against *A. niger* No activity against *A. flavus, A. fumigatous, R. indicum, *and *Fusarium *spp.	(21)
	Whole plant	Diethyl ether	*Candida albicans *and *Aspergillus niger*	Significant growth inhibition against *C. Albicans* and *A. niger*	(27)

**Table 2:** *In vitro* and *in vivo* studies on anti-oxidant and free radical scavenging activities, and hepatoprotective, anti-diabetic, anti-inflammatory, and anti-nociceptive activities of *Hygrophila schulli* plant extract

**Property**	**Extraction**		**Study**	**Test/s**	**Activities/ Remarks**	**Reference/s**
	**Part of the plant**	**Solvent/s**				
Antioxidant and free radical scavenging activity	Seeds	Methanol	*In vitro*	Lipid peroxidation	Active against 5-lipoxygenase (5-LO) Inhibited lipid peroxidation with an IC_50_ value of 20 mg/ml	(38)
	Whole plant	Alcohol		lipid peroxidation, DPPH, and reducing power assay	Terpenoid -rich fraction showed the highest potential to act as an antioxidant and scavenge free radicals	(39)
	Leaves	Water		DPPH, superoxide hydroxyl radical scavenging assay, lipid peroxidation assay	Depicted the highest superoxide radical-scavenging activity	(40)
	Whole plant	Ethanol	*In vivo*	Mercuric chloride-induced oxidative stress	Ethanol extract increased the levels of antioxidant molecule enzymes which protect against oxidative damage	(32)
	Aerial Parts	Alcohol (50%)		Rat liver homogenate	Exhibited good free radical scavenging activity against DPPH and moderate activity against Nitric oxide, hydroxyl radical, ferryl bipyridyl complex, and lipid peroxidation	(41)
Hepatoprotective activity	Seeds	Methanol	*In vivo*	Paracetamol-induced hepatotoxicity	Reduced the level of biochemical parameters (Glutamic oxalacetic transaminase, Glutamic pyruvic transaminase, Alkaline phosphatase, Glutamate dehydrogenase, Serum bilirubin)	(10)
	Whole plant	Water			Hepatic cells significantly regenerated following the treatment of plant extract	(3)
	Seeds	Methanol		Acetaminophen (APAP)-induced hepatotoxicity	Prevented alterations occurring with the use of the drug	(42)
	Whole plant	Water		Carbon tetrachloride-induced hepatotoxicity	Hepatic cells significantly regenerated	(3)
	Root	Water			Increased enzyme level due to liver damage nearing normal with the treatment of plant extract	(43)
					Exhibited protective effect due to its anti-lipid peroxidative and free radical scavenging properties	(33)
		Ethanol			Exhibited protective action in a dose-dependent manner	(44)
	Aerial part	Ethanol		Rifampicin and isoniazid -induced hepatotoxicity	Significantly reduced the biochemical and histological changes induced by the drug	(45)
Anti-diabetic activity	Seeds	Water, ethanol, methanol, and chloroform	*In vitro*	Amylase inhibition studies and glucose diffusion inhibition studies	A methanolic extract found to be a potent anti-diabetic	(36)
	Leaves	Water	*In vivo*	Fasting blood glucose, plasma insulin, hemoglobin, and glycosylated hemoglobin	Significantly decreased the glycosylated hemoglobin, plasma glucose, aspartate transaminase, alanine transaminase, and total serum cholesterol	(46)
	Aerial part	Ethanol (50%)		Fasting blood glucose	Moderately decreased the blood sugar level	(47)
	Whole plant	Water		Fasting blood glucose and glucose tolerance assay	Significantly increased glycogen in the muscles and liver, and triacylglycerol in adipose tissue	(48)
				Glucose oxidase method	Significantly decreased the fasting blood glucose level and markedly improved the glucose tolerance	(49)
Anti-inflammatory activity	Leaves	Ethanol	*In vitro*	Anti-hyaluronidase assay	Tissue permeability was enhanced by the activity of hyaluronidase which spread the inflammatory responses around the affected organ	(4)
	Leaves	Petroleum ether, chloroform and alcohol	*In vivo*	Brewer’s yeast-induced pyrexia and carrageenan-induced paw edema	Chloroform and alcoholic extracts exhibited anti-inflammatory activity	(24)
	Leaves	Chloroform and alcohol		Cotton pellet-induced granuloma	Inhibition of granuloma formation was dose-dependent. The extract exhibited the ability to inhibit the proliferative phase of the inflammation process	(50)
Anti-nociceptive	Leaves, aerial parts, and roots	Chloroform, petroleum ether, Alcohol, and water	*In vivo*	Acetic acid-induced writhing response, tail flick assay, and hot plate reaction time	Exhibited anti-nociceptive activity by both central and peripheral mechanisms	(51)
	Leaves	Petroleum ether, chloroform, alcohol, and water			Exhibited anti-nociceptive activity by both central and peripheral mechanisms	(24)

**Table 3:** *In vivo* studies on anti-cancer, anti-tumor activities, and cytotoxicity of *Hygrophila schulli* plant extract

**Property**	**Extraction/s**		**Cancer type/cell line / experimental model**	**Activity/ Remarks**	**Reference/s**
	**Part of the plant**	**Solvent/s**			
Anti-cancer activity and Cytotoxicity	Seeds	Water and methanol	colon: HT-29 breast; MDA-MB-435S	The methanolic extract had no toxicity against healthy mouse fibroblasts (NIH3T3), but selective cytotoxicity against breast cancer cells (MDA-MB-435S) with IC_50_ of 1.58 mg/ml. Aqueous extract - selective toxicity against colon cancer cell line HT-29 with an IC_50_ of 0.22 mg/Ml	(1)
	Leaves	Hexane, methanol, ethyl acetate, and water	Dalton's Lymphoma Ascites (DLA) and Ehrlich Ascites Carcinoma (EAC) cell lines	Hexane extract gave 98% inhibition against the EAC cell line at 200 μg/ml concentration, aqueous extract resulted in 8% inhibition against the EAC cell line, hexane extract showed 94% inhibition against DLA cell lines at 200 μg/ml aqueous extract gave 7% inhibition against DLA cell lines Methanol, ethyl acetate, and aqueous extract had very low cytotoxic activity against both cell lines	(54)
		Methanol	Liver, kidneys, heart, lungs, spleen & brain of Wister albino female rats	No abnormalities, changes were revealed in the color of the internal organs, presence of lesions, and no acute adverse effects with the treatment of extract at 2000 mg/kg body weight (BW).	(53)
		Ethanol	Swiss albino mice model	Neither mortality nor acute toxicity at a single oral dose of 2 g/kg	(4)
	Whole plant	Hydro-alcohol	Breast carcinogenesis	Significantly reduced the progesterone receptor (ERα, PR) levels and tumor weight	(55)
		Methanol	Breast carcinogenesis	Tumour growth was significantly reduced. Antitumor activity of the extract was dose-dependent	(7)
	Seeds	Methanol	Hepatocarcinogenesis	Antitumor activity of the extract was dose-dependent	(56)

## Conclusion

Based on the available literature evidence, phytoconstituents extracted from various parts of *H. schulli* act as reservoirs of medically important phytochemical agents. Although different parts of the *H. schulli *plant were found to have various medicinal properties against infections and diseases, its anti-biofilm properties are not elucidated yet. Most of the bioactivities of *H. schulli *are due to the presence of phytoconstituents such as flavonoids (ellagic acid, apigenin, quercetin, luteolin, and gallic acid), alkaloids (asteracanthicine and asteracanthine), triterpenes (hentricontane, lupeol, lupenone, and betulin), sterols (asterol and stigmasterol), fatty acids, minerals, aliphatic esters, amino acids, and essential oils. The potential bioactivities, mechanisms of action, chemical profiles, and toxicological assessments of various plant extracts and their fractions provide the necessary information to develop novel therapeutic alternatives with high efficacy, availability, and low toxicity using natural sources. 

## Authors’ Contributions

W, A D, and MM W designed the study; N MC and GK W contributed to writing the Manuscript; R D, MM W, A D, GK W, and S R made contributions in critical revision and final editing of the manuscript.

## Conflicts of Interest

The authors declare no conflicts of interest

## References

[1] Uddin SJ, Grice ID, Tiralongo E (2011). Cytotoxic effects of Bangladeshi medicinal plant extracts. Evid Based Complement Alternat Med.

[2] Tu Y, Yang Y, Li Y, He C (2021). Naturally occurring coumestans from plants, their biological activities and therapeutic effects on human diseases. Pharmacol Res.

[3] Hewawasam RP, Jayatilaka K, Pathirana C, Mudduwa LKB (2003). Protective effect of Asteracantha longifolia extract in mouse liver injury induced by carbon tetrachloride and paracetamol. J Pharm Pharmacol.

[4] Tekulu GH, Desta A, Hiben MG, Araya EM (2020). Anti-nociceptive and anti-inflammatory activity of Hygrophila schulli leaves. J Inflamm Res..

[5] Shoma FK (2018). Phytochemical screening and biological activity evaluation of Hygrophila schulli.

[6] Sufian MA, Haque MR (2015). Cytotoxic, thrombolytic, membrane stabilizing and anti-oxidant activities of Hygrophila schulli. Bangladesh J Pharmacol.

[7] Nair DV, Shridhar NB, Jayakumar K (2015). Evaluation of anticancer activity of Asteracantha longifolia in 7, 12-Dimethylbenz (a) anthracene-induced mammary gland carcinogenesis in Sprague Dawley rats. Int J Nutr Pharmacol Neurol Dis.

[8] Nabèrè O, Adama H, Samson G, Kiessoum K, Patrice Z, Roland MN-T (1930). Antibacterial and phytochemical studies of three Acanthaceae species used in Burkina Faso traditional medicine. J Appl Pharm Sci.

[9] Ahmed S, Riaz M, Malik A, Shahid M (2007). Effect of seed extracts of Withania somnifera, Croton tiglium and Hygrophila auriculata on behavior and physiology of Odontotermes obesus (Isoptera, Termitidae). Biologia (Bratisl).

[10] Singh A, Handa SS (1995). Hepatoprotective activity of Apium graveolens and Hygrophila auriculata against paracetamol and thioacetamide intoxication in rats. J Ethnopharmacol.

[11] Kannur D, Paranjpe M, Dongre P, Kumbhar S, Khandelwal K (2012). Anti-inflammatory and antinociceptive activities of Hygrophila schulli seed extracts. Int J Green Pharm.

[12] Sethiya NK, Ahmed NM, Shekh RM, Kumar V, Singh PK, Kumar V (2018). Ethnomedicinal, phytochemical and pharmacological updates on Hygrophila auriculata (Schum ) Hiene: An overview. J Integr Med.

[13] Chandran RP, Manju S, Vysakhi M V, Shaji PK, Nair GA (2013). In vitro antimicrobial activities of Hygrophila schulli (Buch -Ham) leaf and root extracts against clinically important human pathogens. Biomed Pharmacol J.

[14] Bera S, Das S, Roy A (2017). Ethnobotanical study of Kulekhara (Hygrophila auriculata):A review. Sasta Mukhpatra Annu Tech.

[15] Rahman A, Hossain MM, Islam A (2014). Taxonomy and medicinal uses of angiosperm weeds in the wheat field of Rajshahi, Bangladesh. Front Biol Life Sci.

[16] Islam MS, Parvin MS, Islam ME (2022). The protective and anti-oxidant effects of Hygrophila schulli seeds on oxidative damage of DNA and RBC cellular membrane. Heliyon.

[17] Maia FC, Wijesinghe GK, de Oliveira TR, Barbosa JP, de Feiria SB, Boni GC (2020). Phyllanthus niruri L (stone-breaker) as an alternative of anti-human diseases, antimicrobial agent, and its applicability to combat resistant microrganisms A Brief Review. Braz J Nat Sci.

[18] Wijesinghe GK, Feiria SB, Maia FC, Oliveira TR, Joia F, Barbosa JP (2021). In vitro antibacterial and antibiofilm activity of Cinnamomum verum leaf oil against Pseudomonas aeruginosa, Staphylococcus aureus and Klebsiella pneumoniae. An Acad Bras Cienc.

[19] Wijesinghe GK, Maia FC, de Oliveira TR, de Feiria SNB, Joia F, Barbosa JP (2020). Effect of Cinnamomum verum leaf essential oil on virulence factors of Candida species and determination of the in vivo toxicity with Galleria mellonella model. Mem Inst Oswaldo Cruz.

[20] Oliveira TR, Teixeira AL, Barbosa JP, de Feiria SN, Boni GC, Maia F, Anibal PC, Wijesinghe GK, Höfling JF (2020). Melaleuca spp essential oil and its medical applicability A Brief Review. Braz J Nat Sci.

[21] Esther VCJ, Saraswathi R, Dhanasekar S (2012). In vitro antibacterial and antifungal activities along with x-ray irradiation studies of medicinal plant. Hygrophila auriculata Int J Pharm Pharm Sci.

[22] Boily Y, Van Puyvelde L (1986). Screening of medicinal plants of Rwanda (Central Africa) for antimicrobial activity. J Ethnopharmacol.

[23] Samy RP (2005). Antimicrobial activity of some medicinal plants from India. Fitoterapia.

[24] Patra A, Jha S, Murthy PN, Vaibhav DA (2008). Anthelmintic and antibacterial activities of Hygrophila spinosa T. Anders. Res J Pharm Technol.

[25] Doss A, An SP (2013). Antimicrobial activity of Hygrophila auriculata (Schumac ) Heine and Pergularia daemia Linn. African J Plant Sci.

[26] Vlietinck AJ, Van Hoof L, Totte J, Lasure A, Berghe D Vanden, Rwangabo PC (1995). Screening of hundred Rwandese medicinal plants for antimicrobial and antiviral properties. J Ethnopharmacol.

[27] Hussain AZ, Kumaresan S (2013). GC-MS analysis and antimicrobial activity of Hygrophila auriculata. Arch Appl Sci Res.

[28] Kuruppuarachchi SN, Sandamali SWSP, Sampath MKA, Weerasekara MM, Dilhari KAA (2021). Hygrophila schulli medicinal plant: Phytochemical screening, evaluation of antibacterial activity and minimum inhibitory concentration against Escherichia coli. International Conference on Health Sciences..

[29] Rath S, Bal SCB, Dubey D (2021). Oral biofilm: Development mechanism, multidrug resistance, and their effective management with novel techniques. Rambam Maimonides Med J..

[30] Hamid AA, Aiyelaagbe OO, Usman LA, Ameen OM, Lawal A (2010). Anti-oxidants: Its medicinal and pharmacological applications. African J Pure Appl Chem.

[31] Bagchi K, Puri S (1998). Free radicals and anti-oxidants in health and disease: A review. East Mediterr Health J.

[32] Sridhar MPN, Nandakumar N, Rengarajan T, Balasubramanian MP (2013). Amelioration of mercuric chloride induced oxidative stress by Hygrophila auriculata (K Schum) Heine via modulating the oxidant-anti-oxidant imbalance in rat liver. J Biochem Technol.

[33] Shanmugasundaram P, Venkataraman S (2006). Hepatoprotective and anti-oxidant effects of Hygrophila auriculata (K Schum) Heine Acanthaceae root extract. J Ethnopharmacol.

[34] Chattopadhyay R (2003). Possible mechanism of hepatoprotective activity of Azadirachta indica leaf extract: Part II. J Ethnopharmacol.

[35] Chatterjee TK (2000). Medicinal plants with hepatoprotective properties. Herb options.

[36] Rastogi A, Shankar S, Mahalingam G (2014). Phytochemical screening, anti-oxidant activity and in vitro anti-diabetic activity of aqueous, methanolic, ethanolic and chloroformic extracts of Hygrophila auriculata. Int J Pharm Pharm Sci.

[37] Simon LS (2013). Nonsteroidal anti-inflammatory drugs and their risk: A story still in development. Arthritis Res Ther.

[38] KC SK, Müller K (1999). Medicinal plants from Nepal; II Evaluation as inhibitors of lipid peroxidation in biological membranes. J Ethnopharmacol.

[39] Hussain MS, Ahamed KF, Ravichandiran V, Ansari MZH (2009). Evaluation of in vitro free radical scavenging potential of different fractions of Hygrophila auriculata (K Schum) Heine. Asian J Trad Med.

[40] Dasgupta N, De B (2007). Anti-oxidant activity of some leafy vegetables of India: A comparative study. Food Chem..

[41] Vijayakumar M, Govindarajan R, Shirwaikar A, Kumar V, Rawat AKS, Mehrotra S (2005). Free radical scavenging and lipid peroxidation inhibition potential of Hygrophila auriculata. Nat Prod Sci.

[42] Shivashangari KS, Ravikumar V, Devaki T (2004). Evaluation of the protective efficacy of Asteracantha longifolia on acetaminophen-induced liver damage in rats. J Med Food.

[43] Usha K, Kasturi GM, Hemalatha P (2007). Hepatoprotective effect of Hygrophila spinosa and cassia occidentalis on carbon tetrachloride induced liver damage in experimental rats. Indian J Clin Biochem.

[44] Raju BGS, Battu GR, YB ML (2011). Antihepatotoxic activity of Hygrophila Spinosa roots on CCl4 induced hepatic damage in rats. Pharm Res.

[45] Lina SMM, Ashab I, Ishtiaq Ahmed M, Shahriar M (2012). Hepatoprotective activity of Asteracantha longifolia (Nee ) extract against anti-tuberculosis drugs induced hepatic damage in Sprague-Dawley rats. Pharmacologyonline.

[46] Muthulingam M (2010). Antidiabetic efficacy of leaf extracts of Asteracantha longifolia (Linn ) Nees on alloxan induced diabetics in male albino wistar rats. Int J Pharm Biomed Res.

[47] Vijayakumar M, Govindarajan R, Rao GM (2006). Action of Hygrophila auriculata against streptozotocin-induced oxidative stress. J Ethnopharmacol.

[48] Fernando MR, Wickramasinghe S, Thabrew MI (1998). Extra pancreatic actions of Hygrophila longifolia. Pharm Biol.

[49] Fernando MR, Wickramasinghe SMDN, Thabrew MI, Karunanayaka EH (1989). A preliminary investigation of the possible hypoglycaemic activity of Asteracanthus longifolia. J Ethnopharmacol.

[50] Patra A, Jha S, Murthy PN, Satpathy S (2014). Anti-Inflammatory Activity of Extracts of Leaves of Hygrophila spinosa T. Anders in Chronic Animal Models of Inflammation. , in Proceedings of the 18th International Electronic Conference on Synthetic Organic Chemistry, 1-30 November.

[51] Shanmugasundaram P, Venkataraman S (2005). Anti-nociceptive activity of Hygrophila auriculata (Schum) Heine. African J Tradit Complement Altern Med.

[52] Sung H, Ferlay J, Siegel RL, Laversanne M, Soerjomataram I, Jemal A (2021). Global cancer statistics 2020: GLOBOCAN estimates of incidence and mortality worldwide for 36 cancers in 185 countries. CA Cancer J Clin.

[53] Neharkar VS, Pandhare R (2015). Acute toxicity study of Hygrophila auriculata L leaves methanolic extract in albino rats. J Pharm Chem Biol Sci.

[54] Chandran RP, Manju S, Shaji PK, Nair GA, Sukumar B (2016). In vitro cytotoxic activities of leaf extracts of Thespesia populnea and Hygrophilla schulli against Dalton’s Lymphoma Ascites and Ehrlich Ascites Carcinoma cell lines. Lung Cancer Res.

[55] Pattanayak SP, Sunita P (2008). Antitumor potency and toxicology of an Indian Ayurvedic plant, Hygrophila spinosa. Pharmacologyonline.

[56] Ahmed S, Rahman A, Mathur M, Athar M, Sultana S (2001). Anti-tumor promoting activity of Asteracantha longifolia against experimental hepatocarcinogenesis in rats. Food Chem Toxicol.

[57] Sulaiman CT, Balachandran I (2012). Total phenolics and total flavonoids in selected Indian medicinal plants. Indian J Pharm Sci.

[58] Dhanalakshmi S, Harikrishnan N, Srinivasan N, Pandian P, Tanisha BA, Kumar MT (2020). A perspective overview on Hygrophila auriculata. Pharmacogn J.

[59] Nigam V, Mishra RK, Gupta A, Kumar M (2015). Pharmacognostic study, characterization of marker compounds and pharmacological review of aerial parts of hygrophila auriculata (schumach) heine. World J Pharm Pharmaceutic Sci.

[60] Sanganal J, Suguna R, Ansar Kamran C (2014). Anti-oxidant activity of asteracantha longifolia in dmba induced mammary cancer in sprague dawley rats. J Cell Tissue Res.

